# Renal interstitial cells to the rescue

**DOI:** 10.7554/eLife.86268

**Published:** 2023-02-21

**Authors:** Hannah M Wesselman, Rebecca A Wingert

**Affiliations:** 1 Department of Biological Sciences, University of Notre Dame Notre Dame United States

**Keywords:** zebrafish, kidney, regeneration, renal interstitial cells, PGE2, Zebrafish

## Abstract

The ability of the adult zebrafish to replace damaged nephrons in the kidney depends on renal progenitor cells and renal interstitial cells working closely together.

**Related research article** Liu X, Yu T, Tan X, Jin D, Yang W, Zhang J, Dai L, He Z, Li D, Zhang Y, Liao S, Zhao J, Zhong TP, Liu C. 2023. Renal interstitial cells promote nephron regeneration by secreting prostaglandin E2. *eLife* **12**:e81438. doi: 10.7554/elife.81438.

The fact some animals can regenerate complex parts of their body after injury has captured our imaginations for hundreds of years. For example, multiple fish species – including the goldfish, trout and zebrafish – are able to regenerate their kidneys throughout their lifetime (Reimschuessel, 2001), and the spiny mouse also exhibits striking renal regenerative abilities (Okamura et al., 2021). Humans, on the other hand, lack this power and stop producing nephrons – the functional units of the kidney – about 36 weeks after conception, and can only partially repair damaged nephrons (Romagnani et al., 2013).

In the zebrafish kidney, cells called renal progenitor cells multiply after organ damage and differentiate to make entirely new nephrons (Zhou et al., 2010; Diep et al., 2011; McCampbell et al., 2015). The local environment around progenitor cells helps guide their growth and activity. However, the identity of these nearby cells and how they control regeneration remains unknown. Now, in eLife, Chi Liu, Tao Zhong and Jinghong Zhao and colleagues — including Xiaoliang Liu and Ting Yu as joint first authors – report that these progenitor cells receive their marching orders from other cells that are similar to the renal interstitial cells found in the kidneys of mammals (Liu et al., 2023).

Renal interstitial cells secrete essential factors during development, and also during disease, in mammals (Whiting et al., 1999). However, until this latest work, it was not clear if these cells were also found in zebrafish. To investigate, Liu et al. – who are based at the Army Medical University and East China Normal University – used single-cell sequencing to see if any cells in the zebrafish kidney activate a gene called *fabp10a*, which is highly expressed in renal interstitial cells. This led them to find cells similar to renal interstitial cells nestled amongst nephrons in a tight-knit network in adult zebrafish, and also around clusters of renal progenitor cells after acute injury (caused by exposure to an antibiotic called gentamicin that can lead to toxic reactions in the kidney).

Further analysis revealed that during regeneration, renal interstitial cells significantly upregulate the gene for an enzyme called Cox2a, which is involved in the synthesis of lipid molecules called prostaglandins. Cells often use prostaglandins to communicate with neighboring cells (Figure 1). Liu et al. found that levels of a prostaglandin called PGE2 increased substantially in the regenerating nephron tissue, and that progenitor cells near to the renal interstitial cells expressed a prostaglandin receptor called Ep4b. Genetically or chemically blocking any aspect of this prostaglandin pathway – that is, the production of prostaglandins by Cox2a, the signaling by PGE2 molecules, or the detection of PGE2 via the Ep4b receptor – caused the renal progenitor cells to proliferate less, thus reducing the ability of the nephrons to regenerate.

**Figure 1.**
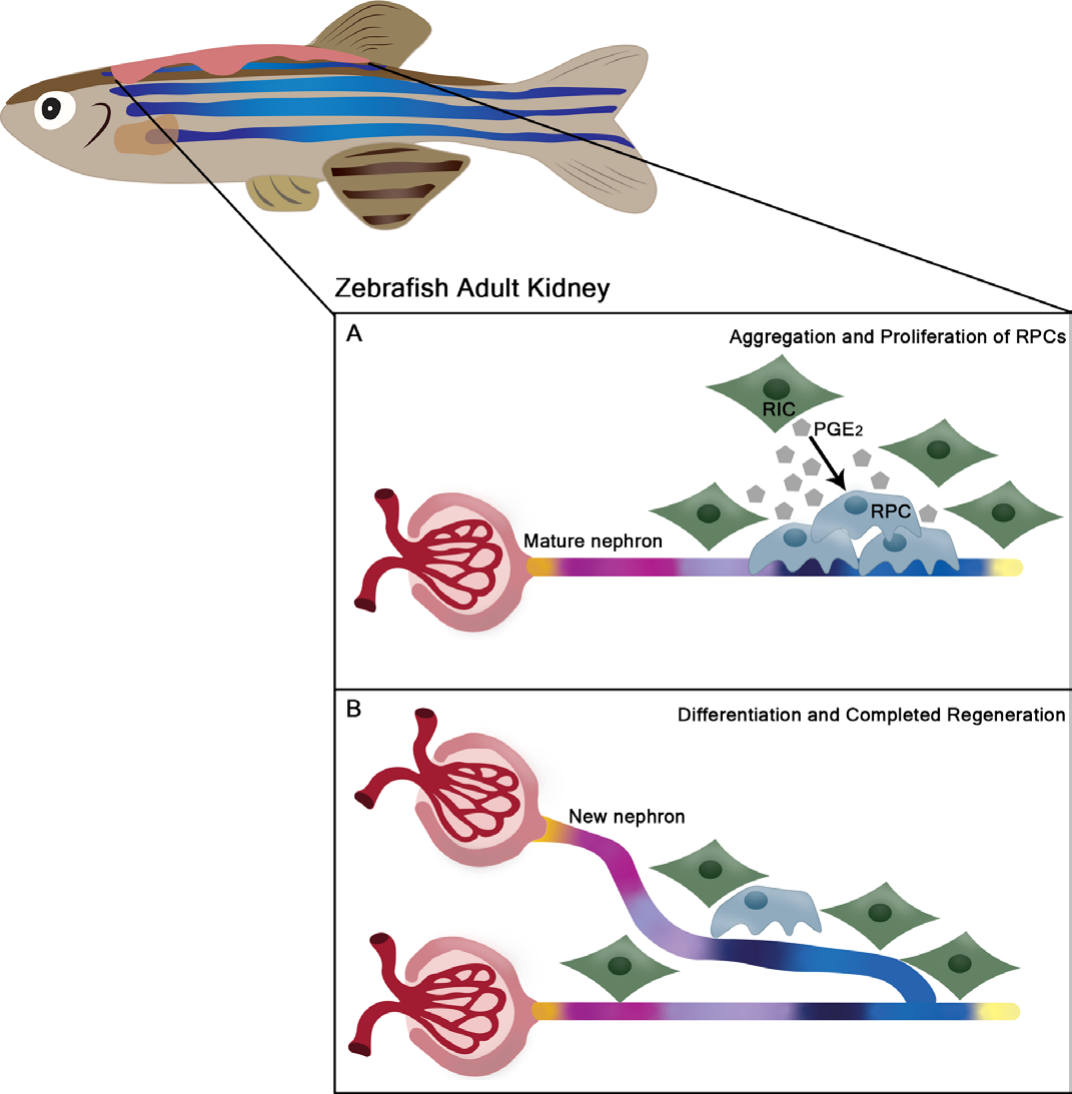
Renal regeneration in the adult zebrafish kidney. (**A**) Following injury, renal progenitor cells (RPCs; blue) in the kidneys of adult zebrafish proliferate and cluster near existing nephrons. This proliferation is promoted by nearby renal interstitial cells (RICs; green) secreting lipid prostaglandin E2 molecules (PGE2; grey polygons), which stabilize a component of the Wnt pathway within the progenitor cells. (**B**) Once these critical signaling events have occurred, the progenitor cells gradually differentiate into other cell types, which go on to form distinct segments of the new nephron (shown as different colors) that replaces the damaged part of the kidney in just a few days.

Image credit: Hannah Wesselman (CC BY 4.0).

PGE2 regulates kidney development in the embryo, as well as the formation of blood stem cells, the liver and other tissues (Poureetezadi et al., 2016; Marra et al., 2019; Jin and Zhong, 2022). To develop these non-kidney tissues, PGE2 works with a well-known signaling network called the Wnt pathway. When the Wnt pathway is inactive, a signaling protein called ß-catenin is routinely produced, but broken down by cellular machinery. When Wnt is active, ß-catenin is not degraded and instead moves to the nucleus and regulates gene expression. Liu et al. found that Wnt signaling was switched on during nephron regeneration, and was diminished when PGE2 signaling was inhibited. Further experiments revealed that addition of PGE2 could rescue ß-catenin stability but not its expression. This suggests that renal interstitial cells in the zebrafish kidney use PGE2 to drive the rapid proliferation of renal progenitor cells by stabilizing the effector of the Wnt pathway.

The findings of Liu et al. reveal how renal interstitial cells are critical for growing new nephrons in the zebrafish kidney, providing valuable insights in to the largely elusive mechanics of renal regeneration. However, questions remain: for example, what triggers cells to synthesize PGE2? Evidence suggests that the gene for Cox2a is not always expressed, implying that dying nephrons somehow trigger its upregulation. Additionally, how does the kidney know when it has reached homeostasis and produced enough new nephrons? While cell proliferation is required for regeneration, too much proliferation is unhealthy – and can even lead to cancer. In other words, now that we understand the components of the regenerative program, how this mechanism is turned off and on again still requires further exploration. Answering these questions will shed new light on the role of the nephron neighborhood during regeneration and, in the future, could help researchers develop better regenerative therapies.

## References

[bib1] Diep CQ, Ma D, Deo RC, Holm TM, Naylor RW, Arora N, Wingert RA, Bollig F, Djordjevic G, Lichman B, Zhu H, Ikenaga T, Ono F, Englert C, Cowan CA, Hukriede NA, Handin RI, Davidson AJ (2011). Identification of adult nephron progenitors capable of kidney regeneration in zebrafish. Nature.

[bib2] Jin D, Zhong TP (2022). Prostaglandin signaling in ciliogenesis and development. Journal of Cellular Physiology.

[bib3] Liu X, Yu T, Tan X, Jin D, Yang W, Zhang J, Dai L, He Z, Li D, Zhang Y, Liao S, Zhao J, Zhong TP, Liu C (2023). Renal interstitial cells promote nephron regeneration by secreting prostaglandin E2. eLife.

[bib4] Marra AN, Adeeb BD, Chambers BE, Drummond BE, Ulrich M, Addiego A, Springer M, Poureetezadi SJ, Chambers JM, Ronshaugen M, Wingert RA (2019). Prostaglandin signaling regulates renal multiciliated cell specification and maturation. PNAS.

[bib5] McCampbell KK, Springer KN, Wingert RA (2015). Atlas of cellular dynamics during zebrafish adult kidney regeneration. Stem Cells International.

[bib6] Okamura DM, Brewer CM, Wakenight P, Bahrami N, Bernardi K, Tran A, Olson J, Shi X, Yeh SY, Piliponsky A, Collins SJ, Nguyen ED, Timms AE, MacDonald JW, Bammler TK, Nelson BR, Millen KJ, Beier DR, Majesky MW (2021). Spiny mice activate unique transcriptional programs after severe kidney injury regenerating organ function without fibrosis. iScience.

[bib7] Poureetezadi SJ, Cheng CN, Chambers JM, Drummond BE, Wingert RA (2016). Prostaglandin signaling regulates nephron segment patterning of renal progenitors during zebrafish kidney development. eLife.

[bib8] Reimschuessel R (2001). A fish model of renal regeneration and development. ILAR Journal.

[bib9] Romagnani P, Lasagni L, Remuzzi G (2013). Renal progenitors: an evolutionary conserved strategy for kidney regeneration. Nature Reviews Nephrology.

[bib10] Whiting PH, Tisocki K, Hawksworth GM (1999). Human renal medullary interstitial cells and analgesic nephropathy. Renal Failure.

[bib11] Zhou W, Boucher RC, Bollig F, Englert C, Hildebrandt F (2010). Characterization of mesonephric development and regeneration using transgenic zebrafish. American Journal of Physiology. Renal Physiology.

